# Host–Guest Chemosensor Ensembles based on Water-Soluble Sulfonated Calix[*n*]arenes and a Pyranoflavylium Dye for the Optical Detection of Biogenic Amines

**DOI:** 10.1021/acs.jafc.3c08695

**Published:** 2024-02-12

**Authors:** Ana Sofia Pires, Kevin Droguett Muñoz, Victor de Freitas, Nuno Basílio, Luís Cruz

**Affiliations:** † REQUIMTE/LAQV, Departamento de Química e Bioquímica, Faculdade de Ciências, Universidade do Porto, Rua do Campo Alegre, Porto 4169-007, Portugal; ‡ REQUIMTE/LAQV, Departamento de Química, Faculdade de Ciências e Tecnologia, Universidade Nova de Lisboa, Monte de Caparica 2829-516, Portugal; § Escuela de Química, Facultad de Química y de Farmacia, Pontificia Universidad Católica de Chile, Santiago 6094411, Chile

**Keywords:** pyranoflavylium dyes, macrocyclic receptors, host−guest complexes, food spoilage, biogenic amines, UV–vis, NMR, fluorescence

## Abstract

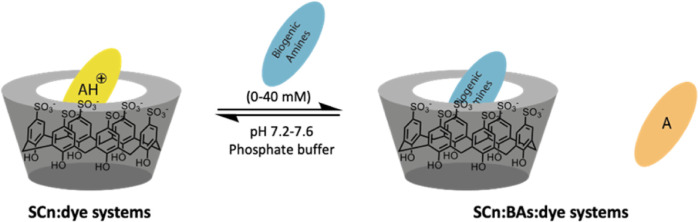

Biogenic amines (BAs) are biologically active nitrogen-containing compounds formed during the food spoilage process and are often related as key markers of food quality, safety, and freshness. Because their presence in foods at high levels can cause significant health problems, researchers have been focused on developing novel strategies and methods for early detection and capture of these analytes. Herein, water-soluble sulfonated calix[*n*]arene macrocycles (SC4, SC6, and SC8) and a pH-sensitive dye (4′-hydroxy-10-methylpyranoflavylium) were investigated as host–guest systems for BA sensing. The hosts were able to bind the flavylium cation of the dye with association constants of 10^3^ to 10^4^ M^–1^. The dye complexation also allowed tuning its p*K*_a_ from 6.72 (free) toward high values: 7.68 (SC4), 7.79 (SC6), and 8.45 (SC8). These data were crucial to optimize the host–guest complexes as optical sensing systems for putrescine/tyramine (pH 7.2–7.6), yielding a colorimetric redshift from yellow to red. The BA sensing was also demonstrated by fluorescence quenching for the calix[*n*]arene/dye complexes and fluorescence recovery after the addition of BAs. ^1^H NMR spectroscopy was used to demonstrate the interaction mode, confirming an encapsulation-driven mechanism. Overall, these host–guest systems demonstrated great potential for the detection of BAs, one of the main key markers of food spoilage.

## 1 Introduction

The development of various synthetic host–guest systems for a wide range of applications, such as smart materials, switches,^1^ catalysis,^2^ sensing,^3−5^ molecular machines, and nanomedicine^6^ have emerged over the last few decades. These complexes are assembled through reversible noncovalent interactions between a host and guest molecule. Typically, the establishment of a host–guest complexation involves more than one type of noncovalent interaction, such as ionic interactions, hydrogen bonding, metal coordination, van der Waals forces, π–π stacking, and solvophobic effects.^7^

Supramolecular chemistry provides a wide range of receptor molecules, particularly macrocycles, that can be used as hosts in a variety of applications, garnering considerable interest from the scientific community. In terms of structural diversity, macrocycles, include cyclodextrins,^8,9^ cucurbiturils,^10,11^ crown ethers,^1^ cryptands,^12^ pillararenes,^13^ and calixarenes,^14,15^ to give some examples. On the other hand, guest molecules are generally small molecules or ions that benefit from the host–guest interactions to improve their stability and bioavailability or even to tune their spectroscopic features, namely, colorimetric and fluorescent properties. Flavylium-based dyes, which includes a vast family of pigments, such as natural anthocyanins, 3-deoxyanthocyanins, and pyranoanthocyanins, exhibit multiple colored species in solution due to their pH-dependent chemical network, being particularly suitable for stimuli-responsive applications.^16^ Due to this property, this type of pigments has been increasingly studied as pH-freshness indicators for applications in food smart packaging.^17−20^

Particularly, pyranoflavylium-based dyes are of great interest and could become very useful as optical guests for the assembly of host–guest complexes with macrocycles. These dyes present a higher chemical stability than their flavylium precursors because of the presence of an additional pyranic ring which blocks the hydration reaction and consequently avoids the ring opening and the formation of the hemiketal and chalcones.^21,22^ Therefore, they can undergo only reversible acid–base reactions producing colored species with different charge-density ratios and spectroscopic features: flavylium cation at low pH, neutral quinoidal base at neutral pH, and anionic quinoidal bases at more basic pH.^23^ Taking into account all of the above features, these pigments offer a significant potential to develop pH-responsive host–guest systems because the presence of structurally diverse chemical species might result in different host affinities.

Several studies focused on chromatographic,^24,25^ electrochemical (amperometric,^26^ voltammetric^27^), and optical sensors to determine BAs. Although each technique has its own advantages, most of them need exhaustive sample preparation procedures and sophisticated equipment that must be operated by highly specialized, trained technicians. In this work, we focus on developing fast and effective optical systems that could be used to evaluate the food spoilage process *in situ*.

The reversibility of host–dye complexes is widely recognized and explored in fields such as switch and sensing systems, including the detection of biogenic amines (BAs).^28−35^ The ability to capture or indicate the presence of relevant analytes associated with food quality, such as BAs, has a wide range of applications in the food industry and particularly in the development of sensing systems to monitor food shelf life in real-time. BAs are physiologically active nitrogen-containing molecules generated during the regular metabolism of animals, plants, and microorganisms.^36^ The presence of these biomolecules in food products is undesirable, and intake of considerable levels can result in headaches, respiratory discomfort, heart palpitations, and a variety of allergic problems.^37^

**Scheme 1:**
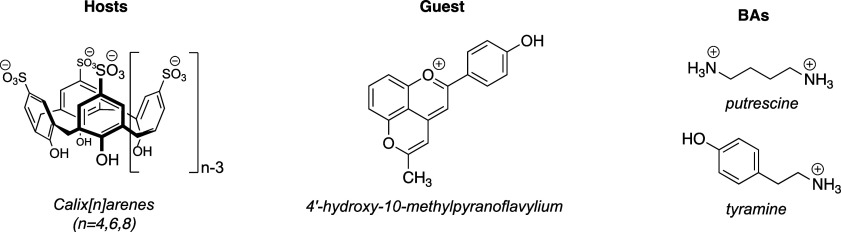
General Chemical Structures of Hosts, Guests, and BAs Used in This Work

The main goal of this work was to develop host–guest supramolecular chemosensors for BA detection based on water-soluble sulfonated-based hosts,^38^ namely, *p*-sulfonatocalix[*n*]arenes (*n* = 4, 6 and 8), and a pyranoflavylium-type pH-responsive guest (4′-hydroxy-10-methylpyranoflavylium dye) (Scheme 1).^39^ In this study, the interaction affinities between the hosts and the different chemical species of the dye in the absence and in the presence of BAs was measured by UV–vis, fluorescence, ^1^H NMR spectroscopy, and isothermal titration calorimetry (ITC).

## 2 Materials and Methods

### 2.1 Materials

2,6-Dihydroxybenzaldehyde, 4-hydroxyacetophenone, chlorotrimethylsilane (TMSCl), acetone, trisodium phosphate (tert) dodecahydrate, and BAs (putrescine and tyramine) were purchased from Sigma-Aldrich. Theorell and Stenhagen universal buffer was obtained as described elsewhere.^40^ The *p*-sulfonated calixarenes (SCn) macrocycles were obtained by ipso-sulfonation of their respective *p*-*tert*-butylcalix[*n*]arenes in sulfuric acid, as previously described.^41,42^

### 2.2 Synthesis of 4′-Hydroxy-10-methylpyranoflavylium Dye (Pyflav)

The synthesis and structural characterization of 4′,5-dihydroxyflavylium was performed according the procedures described in the literature.^22^ Then, 4′,5-dihydroxyflavylium dye was dissolved in an acetone/water (10:90 v/v) solution set at pH 2.9 and 37 °C and after 1 day, the reaction mixture was prepurified by liquid–liquid extraction, as already reported elsewhere.^39^ Afterward, 4′-hydroxy-10-methylpyranoflavylium dye was purified by column chromatography loaded with C18 silica gel, eluted with acid hydroalcoholic solutions containing MeOH 20–30%, and further lyophilized.

### 2.3 UV–vis Host–Guest Titration Experiments

The host–guest association constants were determined for the complexes at pH 1 by UV–visible spectroscopy. For this, a dye solution (0.1 M HCl, 0.75% EtOH) at a concentration of 10 μM was prepared (Solution A) for each host–guest combination. Similarly, a solution containing the dye at the same concentration (10 μM, 0.1 M HCl, 0.75% EtOH) and a known concentration of the host (SC4, SC6, and SC8) was prepared (Solution B). Several solutions containing different concentrations of host (from 0.021 to 3.3 mM) were obtained by adding increasing volumes of solution B to solution A and analyzed through UV–vis spectroscopy. These studies were recorded in a Genesys 105 UV–vis spectrophotometer from 300 to 700 nm, in a quartz cuvette with 1 cm of length path. The fittings for host–guest association constant (*K*_a_) determination were carried out from the least-squares method, using the Solver program from Microsoft Excel.

### 2.4 Determination of p*K*_a_ Values by UV–vis Spectroscopy

Spectrophotometric titrations of the free dye and in the presence of the host (SC4, SC6, and SC8) at fixed concentrations were performed to determine the respective p*K*_a_ values. First, a stock solution of the free dye was prepared in EtOH/H_2_O 75:25 (v/v) with 0.1 M HCl. In a quartz cuvette, 2970 μL of universal buffer (35 × dilution) at desired pH along with 30 μL of the stock solution of the free pigment were added, yielding a solution with EtOH/H_2_O 0.75:99.25 (v/v) and a dye final concentration of 10 μM. In the case of host–guest complexes, the macrocycle amount added was defined according to the previous association constants studies. Titration experiments adding increasing volumes of HCl solution (0.1 M) to the cuvette were performed achieving different pH values, covering the pH range between 10 and 1. After each addition of acid, the mixture was shaken, a UV–vis spectrum was recorded, and the final pH was measured. The p*K*_a_ values were fitted using the least-squares approach and the Solver tool in Microsoft Excel.

### 2.5 pH Measurements

A Radiometer Copenhagen PHM240 pH/ion meter was used to take all pH measurements.

### 2.6 BA Sensing Response

Spectrophotometric titrations of the host–guest complexes were performed to determine the capture ability of the two BAs (putrescine and tyramine). First, a stock solution of free dye was prepared in EtOH/H_2_O 75:25 (v/v) solution with 0.1 M HCl (pH ∼ 1), and a stock solution of putrescine/tyramine in phosphate buffer 10 mM (Na^+^ 10 mM) (pH 7.2–7.6 depending of the macrocycle type). For a quartz cuvette, 2970 μL of phosphate buffer was added at the desired pH, containing a fixed macrocycle amount, and 30 μL of dye stock solution yielding a final solution with 0.75% of EtOH, with a dye final concentration of 10 μM and macrocycles at 2 mM (SC4), 2 mM (SC6), and 1 mM (SC8). Titration experiments were carried out using a putrescine/tyramine stock solution (a phosphate buffer with the same pH value) with increasing quantities added to the cuvette while maintaining the pH constant. After each addition of BA aliquot, the mixture was shaken and a UV–vis spectrum was recorded. The absorbance variation at a fixed wavelength was fitted to achieve the minimum concentration of putrescine/tyramine needed to produce the maximum signal in the host–guest complexes.

### 2.7 Fluorescence Spectroscopy

Fluorescence studies were obtained using a spectrofluorometer FluoroMax-4 (HORIBA Scientific) in a QS high-precision cell made of quartz SUPRASIL with a path length of 3 mm (Hellma Analytics). The concentration of dye solutions was defined as having absorbances equal to or less than 0.1 at the excitation wavelength. For all experiments, the front and exit-entry slits had a 5 nm bandpass, and 440 nm excitation wavelength was chosen for both studied pH values.

### 2.8 ^1^H NMR

^1^H NMR (600.13 MHz) spectra were recorded using a Bruker-Avance 600 spectrometer working at 298.15 K and TSP was used as an internal standard. The three types of samples (Dye, Dye + Host, and Dye + Host + BA) were prepared in D_2_O/MeOD 80:20 at pH 1 and 6.8 with different amounts of acid or base (DCl or NaOD). ^1^H chemical shifts were assigned based on previously published characterization.^21^ Multiplicities are expressed as singlet (s), doublet (d), triplet (t), and chemical shifts (δ) in parts per million, and coupling constants (*J*) in hertz.

### 2.9 ITC Analysis

ITC experiments were carried out on a MicroCal PEAQ-ITC instrument from Malvern. All experiments were conducted at 25 °C in 5 mM phosphate buffer at pH = 7. Typical experiments consist of 19 injections of 2 μL of guest solution (the first injection was 0.4 μL) into the ITC cell containing the calixarene host ca. 10 times more diluted (spacing: 150 s; stir speed: 750 rpm; and injection duration: 4 s). When a plateau was not reached at the end of the titration, the excess of solution was removed from the overflow reservoir, the pipet was refilled with the same guest solution, and a second 19 injections experiment was carried out to complete the titration. The data was concatenated using the MicroCal Concat ITC software. The guest dilution heats were found to be constant and therefore were considered an offset during the data fitting protocol. The data was analyzed by MicroCal PEAQ-ITC analysis software with the one-set-of-sites model, and the first data point from the 0.4 μL injection was always omitted.

## 3 Results and Discussion

### 3.1 4′-Hydroxy-10-methylpyranoflavylium Guest Dye

The synthesis of pyflav pH-sensitive dye was obtained by two reaction steps following the procedures already reported.^39^ Briefly, the 4′,5-dihydroxyflavylium dye was first obtained throughout an acid-catalyzed aldol condensation between 2,6-dihydroxybenzaldehyde and 4′-hydroxyacetophenone in the presence of TMSCl and MeOH to generate gaseous HCl *in situ*.^43^ Following that, pyflav was produced by an annelation reaction between 4′,5-dihydroxyflavylium dye and acetone following the pyranoanthocyanins mechanism described elsewhere.^16^ The dye had been structurally characterized using mono- and bidimensional NMR spectroscopy and mass spectrometry.^39^

### 3.2 Association Constants (*K*)

**Figure 1:**
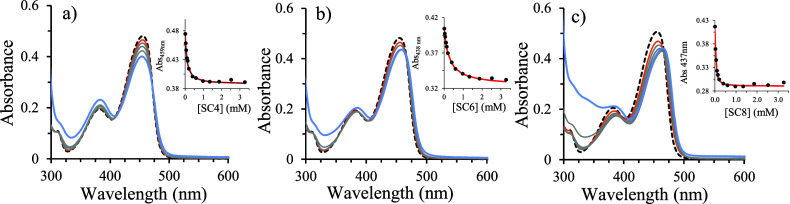
UV–vis absorption spectra of pyflav (10 mM), at pH 1, registered upon the addition of increasing concentrations of (a) SC4, (b) SC6, and (c) SC8. The inset shows the data fitting to a 1:1 binding model. The dashed line represents the free dye, the orange line represents the starting line of the changing trend, the gray lines the successive additions of SCn, and the blue line is the maximum SCn concentration.

As the starting point for this investigation, the binding association constant (*K*) of the pyflav cation with the three sulfonated macrocycles (SC4, SC6, and SC8) was studied using UV–vis absorption/fluorescence at pH 1, where the flavylium cation was the only species present. Figure 1 shows the variations observed in the absorption spectra after the titration of dye with increasing amounts of macrocycle receptors. In all cases, the absorbance spectra exhibited a hypochromic shift and a bathochromic shift (except for the case of SC4) as the amount of host increased.

The absorbance variations of the dye at its maximum wavelength absorption with an increasing macrocycle concentration revealing a binding isotherm that can be fitted to a 1:1 host–guest complexation model. This yielded a first estimate of the binding constant *K* of the pyflav with SC4, which was around 1.3 × 10^4^ M^–1^. The binding affinities of the pyflav cation for the other two sulfonated macrocycles were studied by using identical absorption titration methods. All the titration curves for macrocycle–dye association constant (*K*) determination were fitted to the 1:1 host (H)–guest (G) binding model (see Table 1)
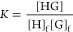
1where |HG| is the concentration of dye complexed and |H|_f_ and |G|_f_ are the concentrations of uncomplexed (free) species in the system.

**Table 1:** Association Constants (*K*) for Pyflav Cations with Three Different Macrocycles at pH = 1

macrocycle	SC4	SC6	SC8
*K* (M^–^^1^) UV–vis	1.34 × 10^4^	4.85 × 10^3^	8.41 × 10^4^
*K* (M^–^^1^) fluorescence	5.93 × 10^3^	3.00 × 10^3^	3.67 × 10^4^

The binding process was monitored by UV–vis spectroscopy and the observed spectrum changes was expressed as
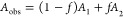
2where *f* is the fraction of dye complexed with macrocycle.

For the expression for free guest ([G]_f_) and complex ([HG]) concentration, we expressed as
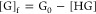
3
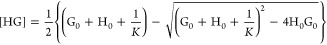
4where G_0_ is the total concentration of guest, H_0_ is the total concentration of host, [HG] is the equilibrium concentration of the host–guest complex, and *K* is the association constant.

**Figure 2:**
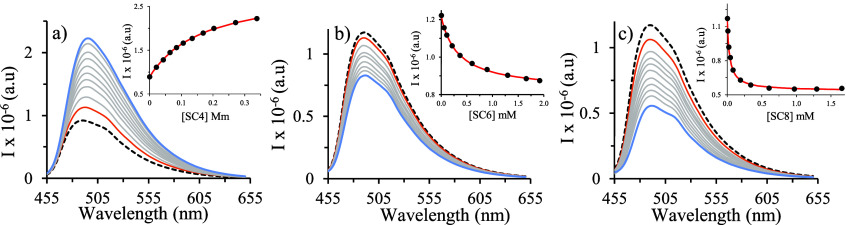
Fluorescence spectra of pyflav (3.2 μM), at pH 1, registered upon addition of increasing concentrations of (a) SC4, (b) SC6, and (c) SC8. The inset shows the data fitting to a 1:1 binding model. The dashed line represents the free dye, the orange line represents the starting line of the changing trend, the gray lines the successive additions of SCn, and the blue line is the maximum SCn concentration.

The host–guest systems were also studied by fluorescence spectroscopy at pH 1. Figure 2 shows the variation in the fluorescence spectra of the dye upon titration with increasing amounts of macrocyclic receptors. In the presence of SC4, the spectral variations revealed a pronounced fluorescence enhancement tentatively assigned to the additional rigidification of the flavylium cation upon bidding by the SC4. However, in the presence of increasing amounts of SC6 and SC8, there was a considerable fluorescence quenching. SCn are known to quench the fluorescence of complexed dyes by excited-state electron transfer from electron-rich phenolic units to the guest. This process is more efficient at neutral pH, where one or more phenolic units are deprotonated, but at acidic pH this process may compete with other phenomena (*e.g.*, confinement) favoring the radiative decay.^44^ The results obtained here suggest that the electron-transfer deactivation efficiency increases from SC4 to SC8, resulting in a decrease of the fluorescence emission efficiency of the dye upon complexation with hexamer and octamer calixarene derivatives.

The binding constants (*K*), obtained at pH 1, which are compiled in Table 1, span from 3.00 × 10^3^ M^–1^ for the SC6 complex to 8.41 × 10^4^ M^–1^ for the complex formed with SC8, being within the range of values previously reported for the complexation of other flavylium compounds with sulfonated calixarenes.^14,45^ Regarding the affinity for the sulfonatocalixarenes, these results suggest that the SCn cavity size and/or the number of sulfonate groups are not the main factors controlling the selectivity, and aspects such as flexibility and conformational structure of the hosts might also have an important influence.

In line with the determination of the association constants, the lowest host concentration to ensure a mole fraction of host–guest complexes higher than 0.8 was also determined and used in all further studies for physical–chemical characterization.

### 3.3 Thermodynamic Parameters (p*K*_a_)

Spectrophotometric titrations were performed to determine the equilibrium acid–base forms and respective acidity constants (p*K*_a_ values) for the free pyflav and host–guest complexes. First, the equilibrium forms of the free pyflav in water was studied by measuring the absorption spectra immediately after a pH jump. There was no evidence of color fading at the different pH, indicating that the hydration process does not occur for this dye, which would yield the formation of hemiketal and chalcone species. Therefore, the research on the chemical equilibria of these systems in solution was focused only on the proton-transfer processes (acid–base equilibria). In fact, identical behavior has been documented for several pyranoanthocyanins previously reported in the literature.^46,47^

The global process is given by the following equations
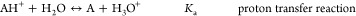
5where
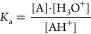
6Considering the total concentration, *C*_0_ is the sum of the individual species
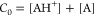
7their mole fraction distribution, χ_i_, is calculated by the simplified equations
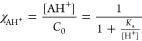
8
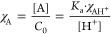
9The fitting of experimental data at a fixed wavelength is given by the following equation
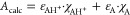
10where ε_AH^+^_ and ε_A_ represent the mole absorption coefficients of individual species.

The apparent p*K*_a_ values were estimated by fitting the absorbance values determined using eq 10 to the ones obtained experimentally at various wavelengths using the Solver add-in program for Microsoft Excel.

**Figure 3:**
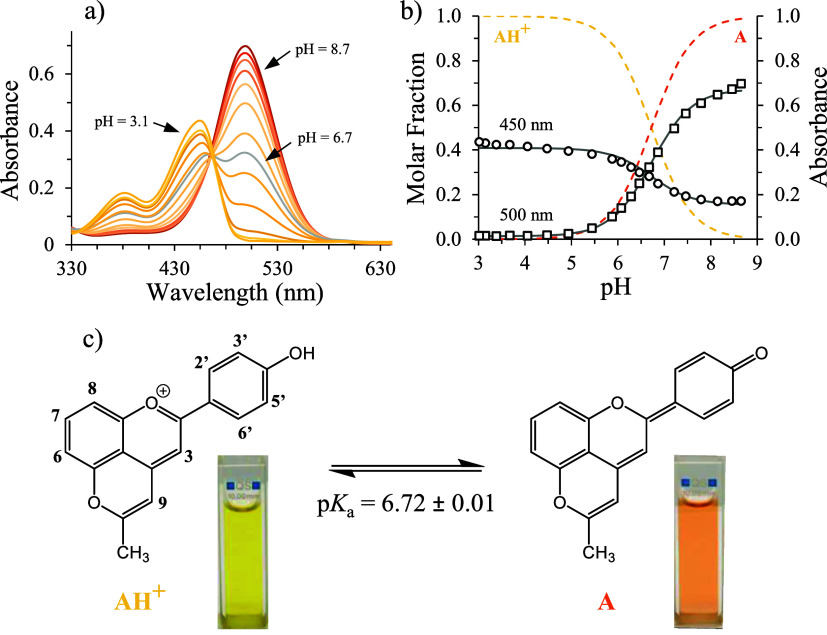
(a) Spectral variations of pyflav as a function of pH; (b) fitting was achieved for p*K*_a_ = 6.72 ± 0.01; and (c) chemical equilibria and species color displayed by free dye in water.

Figure 3a depicts the UV–vis spectral variations of free dye solutions during titration from pH 3 to pH 9. The molar fractions of all species and related absorbance values at two selected wavelengths (450 and 500 nm) are displayed as a function of pH in Figure 3b, along with the corresponding fittings. The p*K*_a_ = 6.72 ± 0.01 is due to the deprotonation reaction of the flavylium species AH^+^ (λ_max_ = 450 nm), giving rise to a neutral quinoidal base A (λ_max_ = 520 nm).

**Figure 4:**
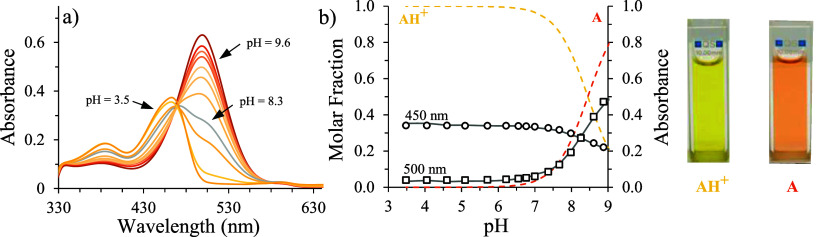
(a) Spectral variations of SC8/dye complex in function of pH and (b) fitting achieved for the p*K*_a_ value (8.45 ± 0.03). All experiments were carried out in a universal buffer (35 × diluted).

The determination of the p*K*_a_ value for the host–guest complexes was also obtained through spectrophotometric titrations using the same experimental conditions for the free dye to ensure direct comparison (Figure 4). Furthermore, this approach was utilized to establish the p*K*_a_ value associated with the deprotonation of the OH group at C4′ in the presence of the host in order to assess whether or not the interaction/encapsulation may tune/shift the p*K*_a_ value compared to the free dye.

While the complexation process to sulfonated calixarenes (SC4, SC6, and SC8) did not considerably shift the absorption maximum wavelength of each species with respect to that of the free dye, the interaction resulted in a very significant increase in the p*K*_a_ value, as shown in Table 2. The most significant variation was observed for the SC8-dye complex. Upward p*K*_a_ shifts are frequently observed for sulfonated calixarene-based host–guest complexes, including flavylium cations, as a result of the selectivity of these receptors toward positively charged species.^45,48,49^

**Table 2:** p*K*_a_ Values Obtained for the Free Dye and for the Three Host–Dye Complexes

	free dye	SC4	SC6	SC8
p*K*_a_	6.72 ± 0.01	7.68 ± 0.02	7.79 ± 0.02	8.45 ± 0.03

### 3.4 BA Sensing by Host–Guest Systems

To assess the host–guest systems’ ability to recognize and detect biomolecules such as BAs, spectrophotometric/fluorescence indicator displacement assays^5,50,51^ with increasing concentrations of those analytes were performed. Putrescine and tyramine were used as model compounds in this study to better understand the behavior of host–guest chemosensors upon exposure to different small molecules with considerable structural variations. The results obtained from the optical experiments were further supported by ^1^H NMR and ITC experiments. First, the pH value for this investigation was fixed and set to the range between the p*K*_a_ of the free pigment to those of host–guest complexes to optimize the optical response of the chemosensors.

#### 3.4.1 ITC Analysis

**Figure 5:**
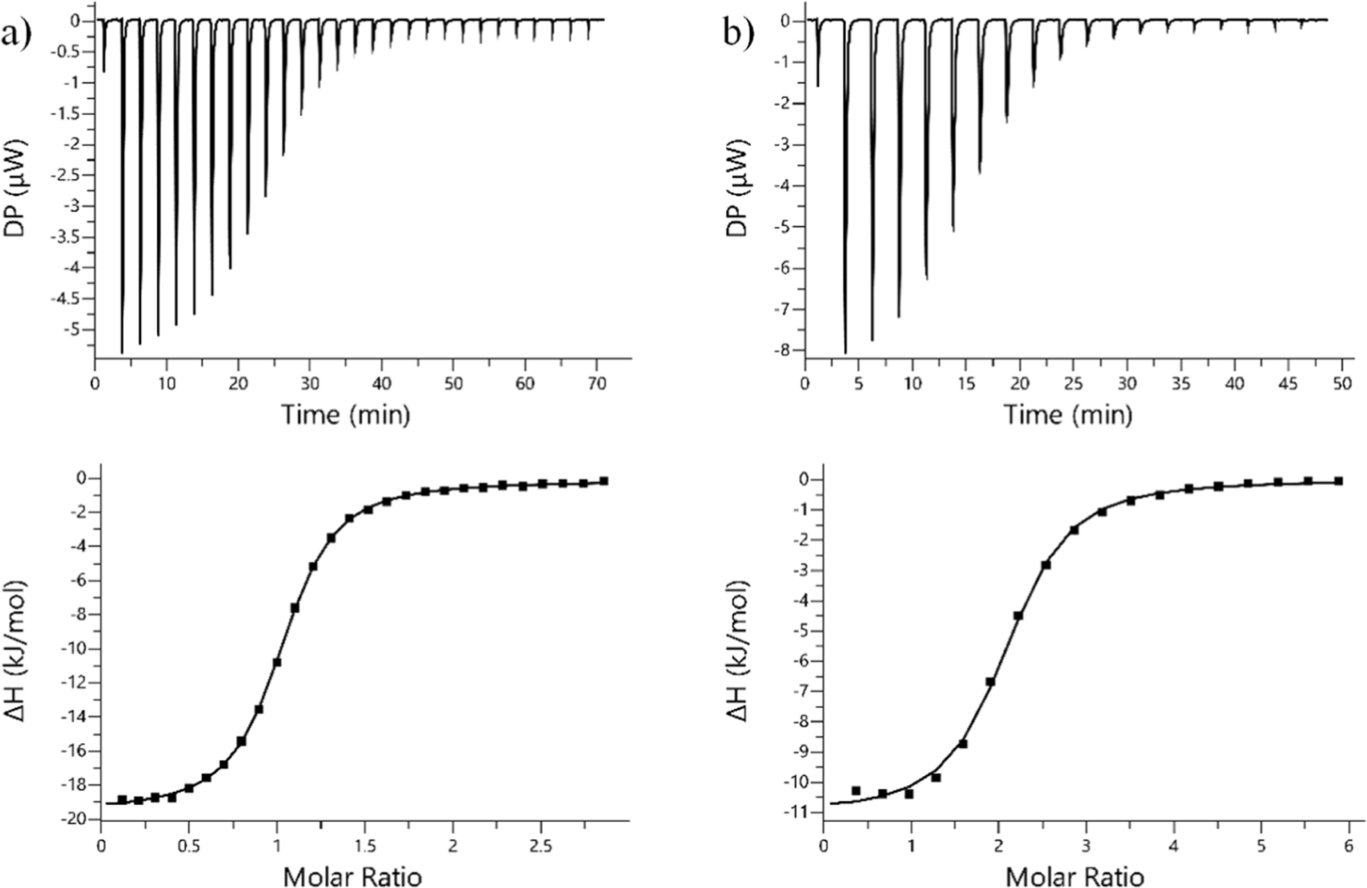
ITC isotherms for the (a) titration of putrescine (1.43 mM) into a solution of SC4 (150 μM) and for the (b) titration of putrescine (4.0 mM) into a solution of SC8 (131 μM). Both titrations were performed in 5 mM of phosphate buffer (pH = 7.2) at 25 °C.

The binding of putrescine and tyramine toward SC4, SC6, and SC8 was investigated by ITC in 5 mM of phosphate buffer at pH = 7.2 to keep the conditions similar to those employed in the indicator displacement assays. It is worth noting that, considering the known competitive binding of sodium ions toward SCn, the thermodynamic parameters obtained from these experiments correspond to apparent values that are dependent on the concentration of these cations.^52,53^Figure 5 shows two representative ITC examples obtained for the titration of SC4 and SC8 with putrescine (see the Supporting Information for the remaining ITC data). The ITC results indicate that SC4 and SC6 form 1:1 host–guest complexes with both amines, while the larger is able to bind two guest molecules simultaneously. Fitting the ITC data to the one set of binding site models (*n* = 1 for SC4 and SC6, *n* = 2 for SC8) allows the direct determination of the apparent binding constants and binding enthalpies, which can be combined to calculate the binding entropies. These thermodynamic data are summarized in Table 3. As can observed, SC4 presents the highest affinity toward putrescine and as well the highest selectivity for this diamine over tyramine. It is also worth noting that SC6 is the less effective binder for putrescine, while SC8 shows the highest affinity for tyramine. The obtained thermodynamic parameters show that the formation of SCn/putrescine host–guest complexes is entropy and enthalpy driven. The significantly favorable entropic changes together with the lower enthalpic variations observed for this highly charged guest suggest that, in addition to the expected noncovalent contacts, there is a significant contribution associated with the release of hydration water molecules upon host–guest association to the overall thermodynamic driving force. In contrast to putrescine, the binding of tyramine to SCn is completely enthalpy driven in line with previous thermodynamic data reported for the binding of aromatic ammonium guests to SCn.^54−56^

**Table 3:** Thermodynamics Parameters for the Formation of Host–Guest Complexes of SCn with Putrescine (PUT) and Tyramine (TYR)^a^

		*K*/M^–^^1^	Δ*H*/kJ mol^–^^1^	–*T*Δ*S*/kJ mol^–^^1^	Δ*G*/kJ mol^–^^1^	*n*
SC4	PUT	2.91 × 10^5^	–19.4	–11.9	–31.2	1
	TYR	1.02 × 10^3^	–27.6	10.4	–17.2	1
SC6	PUT	7.58 × 10^4^	–13.1	–14.7	–27.9	1
	TYR	1.08 × 10^3^	–30.7	13.3	–17.3	1
SC8	PUT	1.17 × 10^5^	–11.1	–17.9	–29.0	2
	TYR	2.37 × 10^4^	–26.1	1.1	–25.0	2

a All experiments were carried out in phosphate buffer at pH 7.2.

#### 3.4.2 UV–vis Spectroscopy

**Figure 6:**
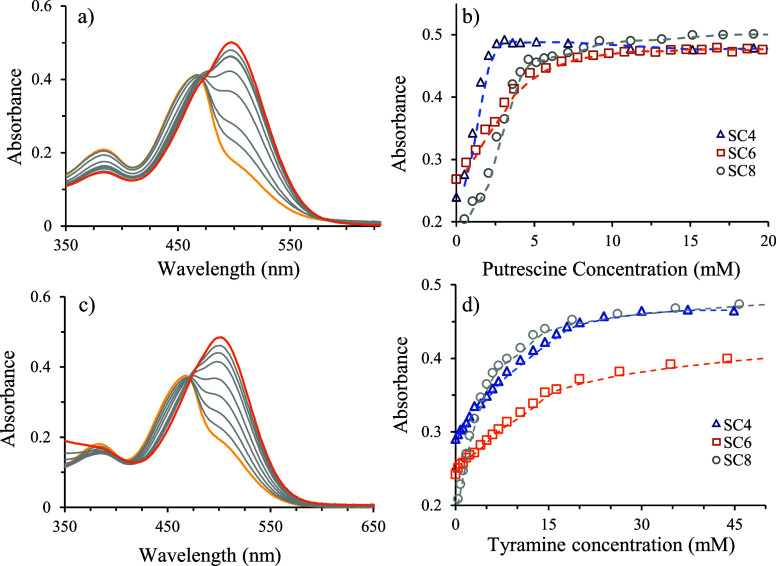
(a) Spectral variations of SC4/dye complex resulting from the addition of putrescine at pH 7.2; (b) variation of the absorbance of SCn/dye complexes at 500 nm with increasing amounts of putrescine; (c) spectral variations of SC8/dye complex as a function of increasing amounts of tyramine at pH 7.6; and (d) variation of the absorbance at 500 nm of SCn/dye complexes with increasing amounts of tyramine. All experiments were carried out in phosphate buffer (10 mM).

The colorimetric response ability of host–guest systems to the presence of BAs in solution was assessed. To accomplish this, increasing concentrations of BAs were added to a solution containing pigment (10 μM) and predefined host concentrations. All studies were conducted in phosphate buffer (10 mM) to preserve the pH value during the putrescine/tyramine additions. The addition of increasing quantities of BAs to the SCn/dye complexes yielded significant absorbance spectral variations (Figure 6).

In all three systems, the spectra displayed a bathochromic shift when the quantity of BA present increased (the spectral variations of SC6 dye/SC8 dye due to putrescine addition and SC4 dye/SC6 dye as a function of increasing tyramine concentration are presented in the Supporting Information). Because the pH was kept constant during the putrescine/tyramine titration, the observed effect was safely assigned to the competitive dissociation of the SCn/dye complex. Owing to the higher p*K*_a_ of the complex, the release of the pigment from the complex to the bulk by competitive BA binding results in the gradual appearance of the neutral quinoidal base species of the dye. Plotting the absorbance variations at 500 nm against the concentration of BAs (Figure 6b) shows that the SC4/dye system presents the highest signal response at lower concentrations of putrescine compared to tyramine. As anticipated from the lower affinities of these calixarenes toward tyramine, higher concentrations of this BA are required to dissociate the SCn/dye reporter pairs, with SC8 offering a better signal response for the detection of tyramine. As expected, these observations are in line with the binding affinities of the investigated BAs for the SCn through ITC. To clarify this behavior, we evaluate the limit of detection (LoD) and the limit of quantification (LoQ) of the three SCn/dye self-assembled chemosensors in the presence of putrescine and tyramine (see Table S1). The obtained LoD and LoQ values were 5 to 18 times higher for tyramine than putrescine, showing that the SCn/dye systems are more sensitive toward putrescine. The observed differences in the sensitivity limit of putrescine versus tyramine can be related to the dicationic nature of putrescine, which displays a higher affinity than the monocationic tyramine toward the negatively charged calixarene hosts.

#### 3.4.3 Fluorescence Spectroscopy

Both direct host–guest and competitive titrations monitored by fluorescence spectroscopy were carried out to evaluate the application of this technique for the detection of BAs in aqueous solutions.

All experiments were carried out in phosphate buffer to maintain the same pH value used in UV–vis experiments during titrations.

**Figure 7:**
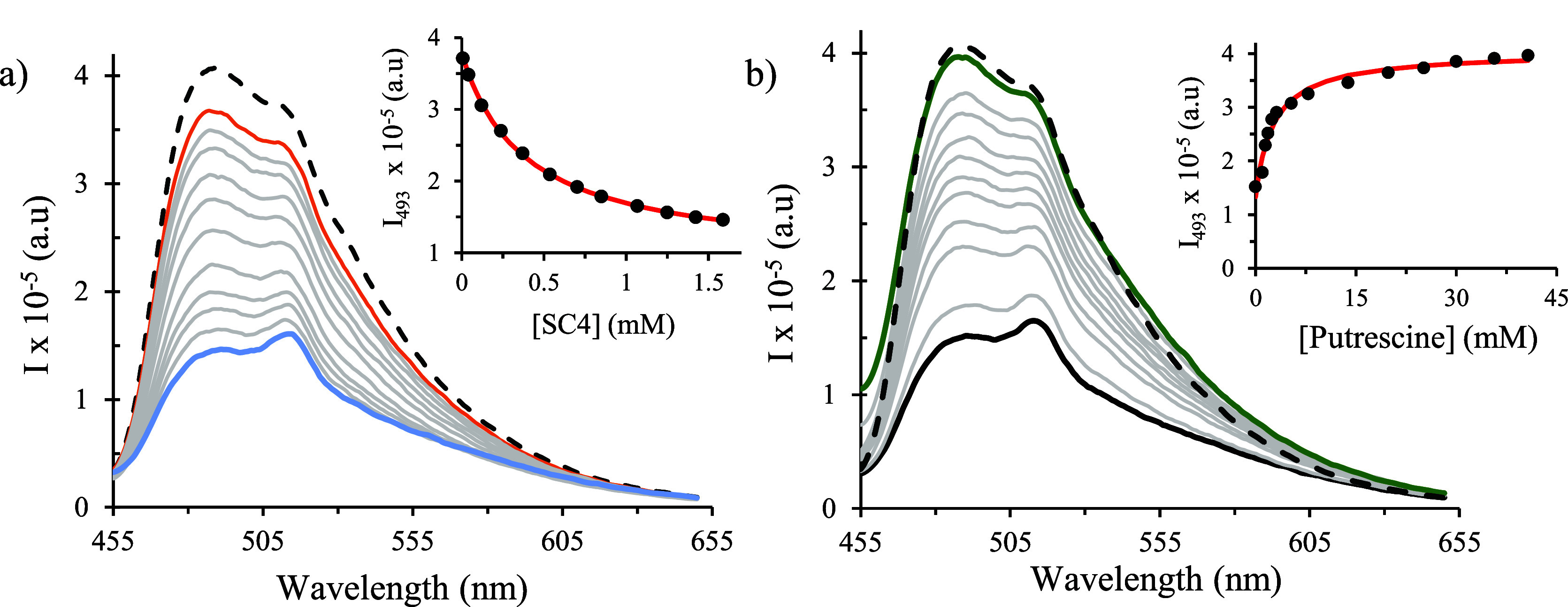
(a) Fluorescence spectra of dye (3.2 μM) titration with increasing concentrations of SC4 with the inset showing the data fitting to an appropriate binding model. The dashed line represents the free dye, the orange line represents the starting line of the changing trend, the gray lines is the successive additions of SC4, and the blue line is the maximum concentration of SC4. (b) Spectral variation of dye (3.2 μM) and SC4 (0.70 mM) with increasing amounts of putrescine at pH 7.2. The black line represents the SC4/dye complex at a molar ratio of 1:0.005, the gray lines represent the successive additions of putrescine, the green line represents the highest putrescine concentration, and the dashed line represents the free dye for comparison.

Figure 7a depicts the fluorescence spectral variations after the titration of the dye with increasing amounts of sulfonated receptor SC4. The intrinsic fluorescence displayed by the free dye is due to the flavylium cation **AH**^**+**^ species because the neutral quinoidal base **A** does not present a radiative emission (see the Supporting Information). When SC4 was added at pH 7.2, a fluorescence quenching was observed and fitted through a 1:1 host (H)–guest (G) binding model. The fluorescence quenching is in line with what is generally observed for dyes bound to SCn due to electron transfer from the phenol/phenolates units to the excited states.^57^ Then, the addition of known amounts of putrescine results in a significant fluorescence increase, suggesting that the SC4/putrescine complexation leads to the release of the dye to the bulk (Figure 7b). The addition of a large excess of putrescine results in the recovery of the dye fluorescence with similar intensity to the one obtained for the free dye solution. For the titration with SC6, the behavior was similar (see the Supporting Information).

On the other hand, upon the addition of increasing concentrations of SC8 receptor to the dye solution, a fluorescence quenching was observed until 0.2 mM of SC8 and then an increase in the fluorescence intensity with λ_max_ of 513 nm was observed. This titration data can be fitted to a host–guest 1:2 binding model, which accounts for the coexistence of HG and HG_2_ complexes (see the Supporting Information). Afterward, the response of the SC8-dye complex to increasing concentration of putrescine demonstrates a significant fluorescence increase of the system overtaking the intensity of the free dye, suggesting that the dye is not (completely) released to the bulk, which might indicate the formation of heteroternary SC8/dye/putrescine 1:1:1 complexes.

### 3.5 NMR Spectroscopy

In order to clarify the interaction and complexation modes established between the dye, macrocycles, BAs, and ^1^H NMR spectroscopy was used.

#### 3.5.1 SCn/Dye Systems

First, the interaction of the SCn/dye complexes was evaluated by ^1^H NMR experiments, conducted at pH 1 with a fixed concentration of dye and increasing concentrations of the hosts (SCn). The presence of SCn leads to a significant complexation-induced chemical shift change to a higher field (Δδ < 0) in ^1^H NMR signals of the dye, as presented in Table 4, which supports the formation of host–guest complexes.

**Table 4:**
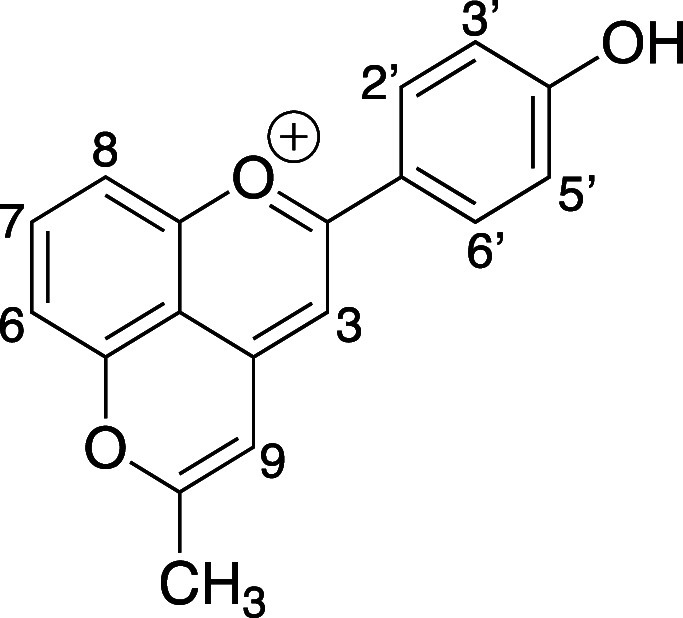
Complexation-Induced Chemical Shift (Δδ) of Guest Protons Observed in the Sulfonated Calixarenes/Pyflav 1:1 Complexes by ^1^H NMR Spectroscopy in DCl 0.1 M

host	Δδ (ppm)							
	H7	H2′/6′	H6 or H8		H3	H3′/5′	H9	CH_3_
**SC4**	–1.58	–0.02	–1.10	–1.40	–0.15	–0.02	–0.49	–0.80
**SC6**	–0.48	–0.09	–0.29	–0.40	–0.18	–0.06	–0.40	–0.37
**SC8**	–0.64	–0.33	–0.57	–0.59	–0.67	–0.22	–1.00	–0.53

**Figure 8:**
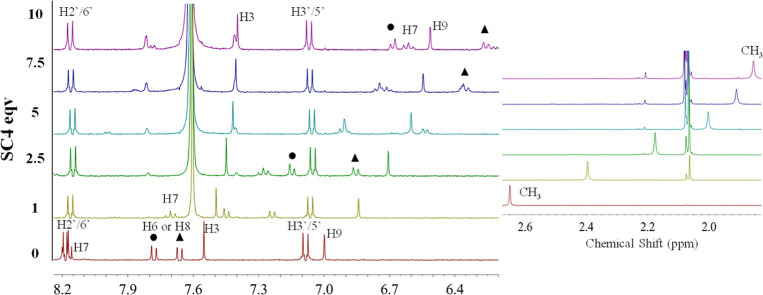
^1^H NMR spectra variations of dye (0.3 mM) solution upon titration with increasing concentrations of the host SC4. The spectra were acquired in D_2_O/MeOD (80:20) at pH 1 with TSP as the internal standard. The protons were labeled according to Figure 3.

The most affected ^1^H NMR signals upon complexation with the SC4, as shown in Figure 8a, were H6, H7, and H8, which are located on the A ring of the flavylium backbone, suggesting that the A ring of the dye is strongly oriented toward the macrocycle cavity. A substantial shielding effect was also observed for H9 and CH_3_, indicating that the D ring moiety might also play an important role in the interaction within the cavity of this macrocycle. Oppositely, H3′/5′ and H2′/6′ protons, from ring B, maintained their chemical shift practically unaltered during the titration, indicating that this ring is probably exposed to the solvent and outside the receptor cavity.

In the complex with SC6, as shown in Figure S8a, the behavior of the guest’s A and D ring protons is similar to the previously described SC4 complex.

Regarding the SC8/dye complex, as shown in Figure S8b, the main difference is the relevant upfield chemical shift observed for the B ring protons, namely, H3′/5′ and H2′/6′. This phenomenon suggests that in this case the dye is more available to penetrate within the macrocycle hydrophobic cavity, indicating the importance of cavity size on the dye encapsulation.

#### 3.5.2 Effect of Putrescine Addition to SCn/Dye Systems

The effect of adding putrescine to the SCn/dye complexes was examined at optimal interaction pH (7.2 for SC4 and SC6 and pH 7.6 for SC8), also through ^1^H NMR experiments.

**Figure 9:**
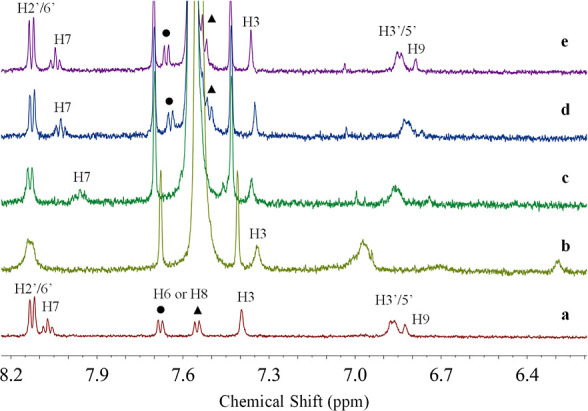
Part of the ^1^H NMR spectra of (a) free dye (0.3 mM), (b) dye (0.3 mM) with SC4 (0.74 mM), and (c–e) SC4/dye at the same ratio of with increasing concentration of putrescine (0.74, 1.86, and 3.72 mM, respectively). All spectra were acquired in D_2_O/MeOD (80:20) at pH 7.2 with TSP as the internal standard. The protons were labeled according to Figure 3.

As shown in Figure 9, the addition of SC4 to free pigment, at pH 7.2, causes the disappearance of several guest protons, in particular, H6/7/8, as well as a clearly significant upfield shift of the H9 proton. It appears that there is a decrease in signal resolution, which is most likely due to the fact that we are dealing with a flavylium/quinoidal base mixture at this pH. Nonetheless, the similarity of the findings that were reported at pH 1, suggesting that complexation occurs mostly by encapsulation of the guest molecule, primarily by rings A and C. However, with the addition of the putrescine, the guest protons began to move downfield, which is consistent with the dye being released from the host cavity, concomitantly switching toward the interaction with the BA. Furthermore, it was possible to observe the reappearance of the H7, H6, and H8 protons, indicating the successful dye dissociation from the host upon BA capture. The SC6 system has a similar behavior to what was previously described for the SC4/dye complex.

**Figure 10:**
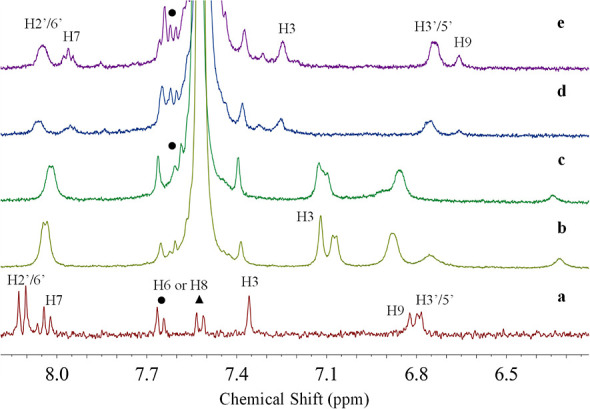
Part of the ^1^H NMR spectra of (a) free dye (0.3 mM), (b) dye (0.3 mM) with SC8 (0.45 mM), and (c–e) SC8/dye at the same ratio of (b) with increasing concentration of putrescine (2.25, 3.37, and 4.75 mM, respectively). All spectra were acquired in D_2_O/MeOD (80:20) at pH 7.6 with TSP as the internal standard. The protons were labeled according to Figure 3.

The interaction of the SC8/dye complex with putrescine was studied at pH 7.6, as shown in Figure 10. The addition of SC8 to the free dye (Figure 10b) resulted in changes identical to those previously described for SC4 and SC6, but we identified a significant upfield shift of the H3 proton. These differences may imply that dye encapsulation, within the macrocycle cavity, is more effective in this complex (SC8/dye), which is explained by the larger cavity and also corroborated by the same observed behavior when we evaluated this system at pH 1. Figure 10c–e depicts the increasing addition of putrescine to the SC8/dye complex, and we noticed that the guest protons began to shift downfield and reappear (H7 and H6), which is consistent with the dye being released from the host hydrophobic cavity and switching to the interaction with the BA.

Host–guest systems based on water-soluble sulfonated calix[*n*]arenes and a pyranoflavyllium-type dye were developed to detect BAs in solution. In this work, the interaction between a previously reported pyranoflavyllium dye and sulfonated calix[*n*]arenes receptors with different sizes and cavities (SCn, *n* = 4, 6, 8) were investigated yielding different binding constants and resulting significant changes in the dye p*K*_a_. Several techniques (UV–vis, fluorescence, NMR, and ITC) allowed us to demonstrate that the interaction between the host-dye complexes can be explored to develop indicator displacement assays for the detection of BAs. In the presence of BAs, the guest molecule could be released or dissociated from the host, and subsequently, the BA could be captured or detected, yielding both colorimetric and fluorescence responses. Therefore, these systems showed great potential for application in food matrixes for the detection of one of the most common food spoilage markers.

## Supporting Information Available

ITC isotherm titrations of BAs with SCn hosts; spectral variations of SCn-dye systems with BAs additions; LoD and LoQ values of BAs for SCn-dye systems; fluorescence spectra titration of dye with SC6 and SC8 hosts; fluorescence spectra of dye at pH 1 and pH 8.5; and ^1^H NMR spectra titration of dye with SC6 and SC8 hosts (PDF)
