# Supplementary material for: Host–Guest Chemosensor Ensembles based on Water-Soluble Sulfonated Calix[n]arenes and a Pyranoflavylium Dye for the Optical Detection of Biogenic Amines
Source: J Agric Food Chem. 2024 Feb 12;72(7):3719–29. doi: 10.1021/acs.jafc.3c08695 (PMC10885154; doi:10.1021/acs.jafc.3c08695)
Supplement: Supplementary file 1 — jf3c08695_si_001.pdf [file jf3c08695_si_001.pdf]

Supporting Information

Host-guest chemosensor ensembles based on water-soluble sulfonated calix[n]arenes and a pyranoflavylium dye for the optical detection of biogenic amines

Ana Sofia Pires,[a] Kevin Droguett Muñoz,[b,c] Victor de Freitas,[a] Nuno Basílio,[b,*] Luís Cruz[a,*]

[a]*REQUIMTE/LAQV, Departamento de Química e Bioquímica, Faculdade de Ciências, Universidade do Porto, Rua do Campo Alegre, 4169-007, Porto, Portugal.*

[b]*REQUIMTE/LAQV, Departamento de Química, Faculdade de Ciências e Tecnologia, Universidade Nova de Lisboa, 2829-516 Monte de Caparica, Portugal.*

[c]*Escuela de Química, Facultad de Química y de Farmacia, Pontificia Universidad Católica de Chile, 6094411 Santiago,Chile*

*Email: luis.cruz@fc.up.pt; nuno.basilio@fct.unl.pt

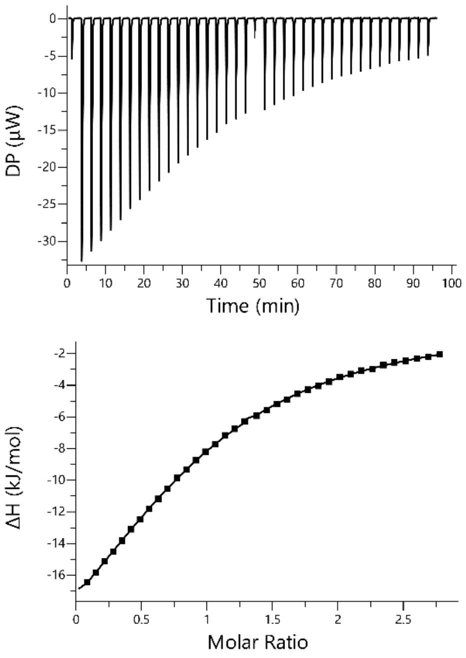

Figure S1: ITC isotherms for the titration of tyramine (10 mM) into a solution of SC4 (1.5 mM) in 5 mM of phosphate buffer (pH = 7.2) at 25 °C.

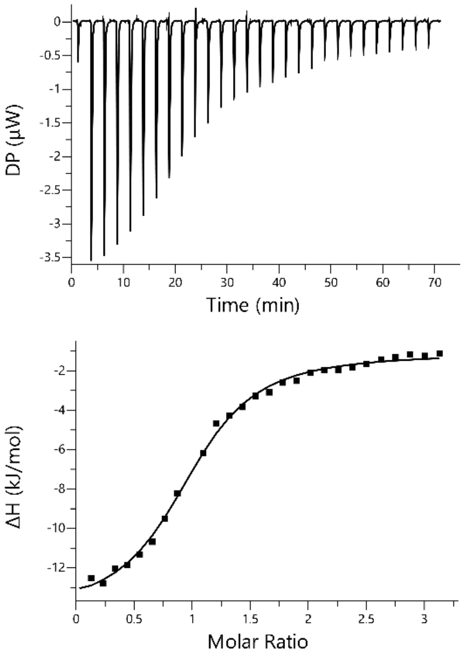

Figure S2: ITC isotherm for the titration of putrescine (1.43 mM) into a solution of SC6 (137 μM) in 5 mM of phosphate buffer (pH = 7.2) at 25 °C.

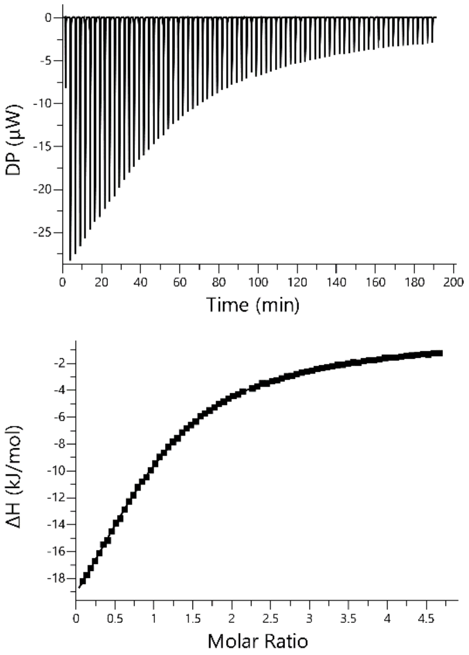

Figure S3: ITC isotherm for the titration of tyramine (14.8 mM) into a solution of SC6 (1.37 mM) in 5 mM of phosphate buffer (pH = 7.2) at 25 °C.

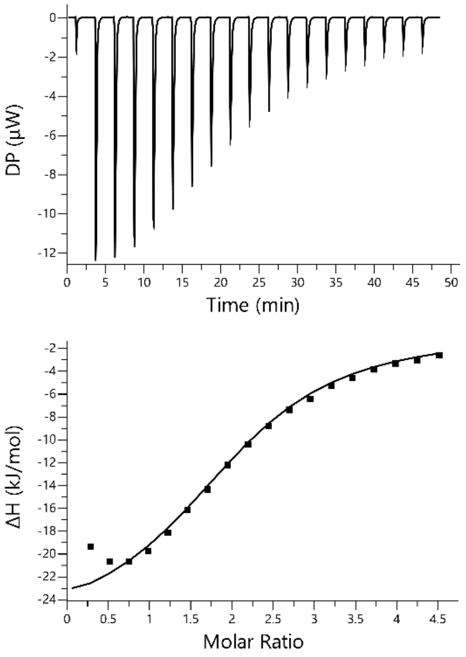

Figure S4: ITC isotherm for the titration of tyramine (3.07 mM) into a solution of SC8 (131 µM) in 5 mM of phosphate buffer (pH = 7.2) at 25 °C. The first 2 signals were omitted for the fit.

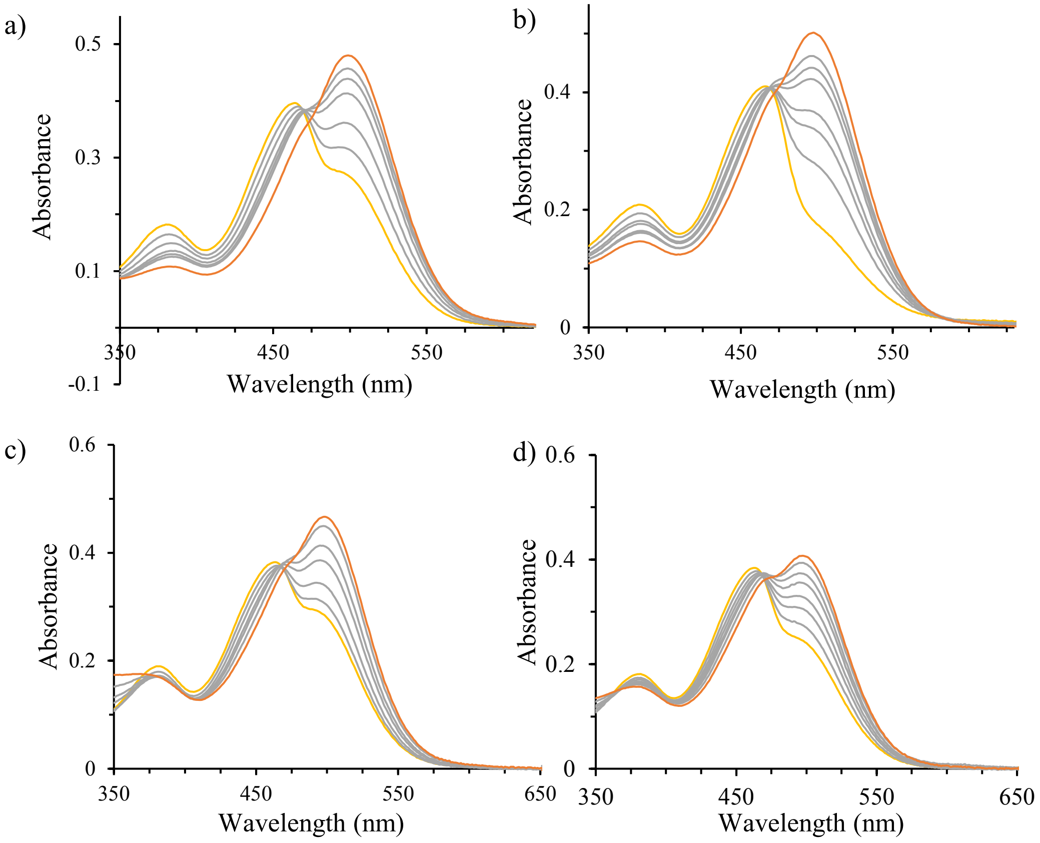

Figure S5: a) Spectral variations of SC6-dye resulting from the addition of putrescine at pH 7.2; b) Spectral variations of SC8-dye resulting from the addition of putrescine at pH 7.6; c) Spectral variations of SC4-dye resulting from the addition of tyramine at pH 7.2 and d) Spectral variations of SC6-dye resulting from the addition of tyramine at pH 7.2.

Table S1 summarizes the LoD and LoQ values for the detection of putrescine and tyramine, for each SCn-dye system.

	**Putrescine**		**Tyramine**	
	**LoD (mM)**	**LoQ (mM)**	**LoD (mM)**	**LoQ (mM)**
**SC4**	0.0793	0.264	1.47	4.90
**SC6**	0.350	1.17	1.79	5.97
**SC8**	0.0260	0.085	0.19	0.62

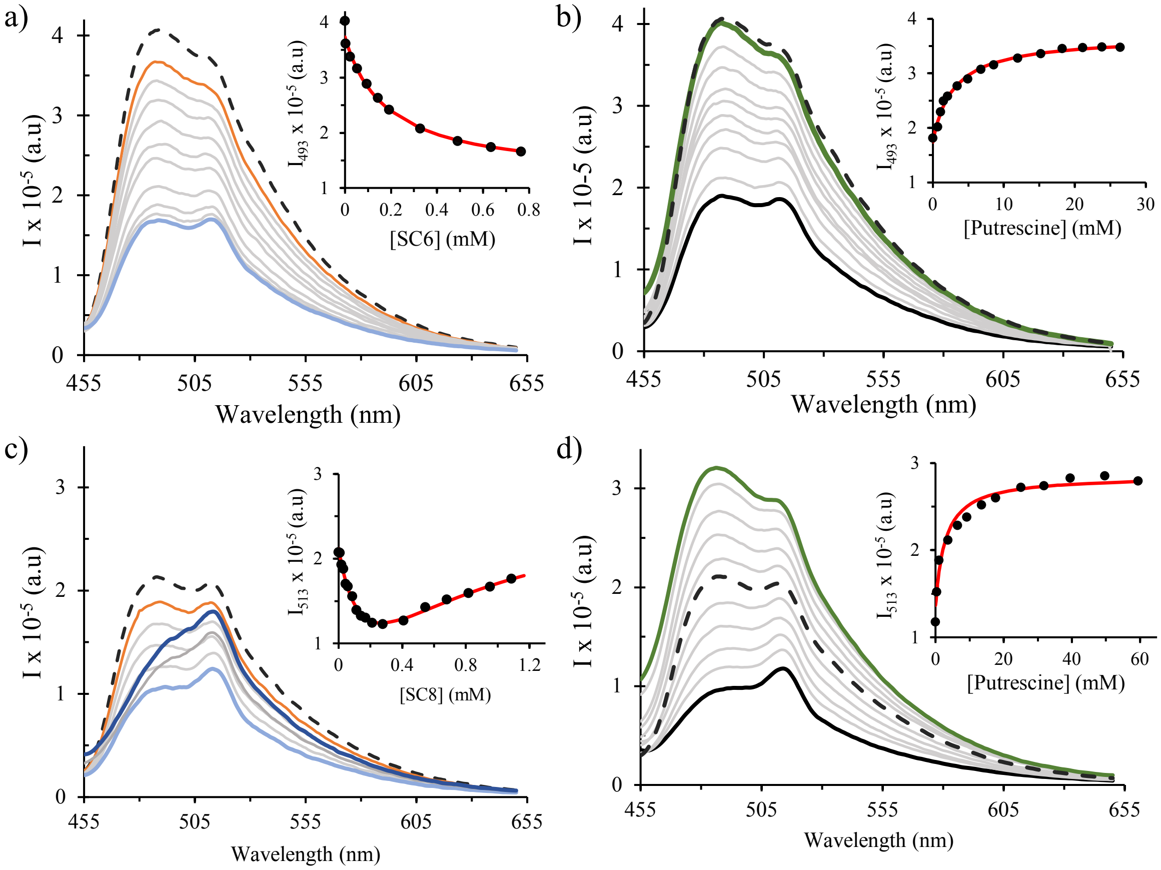

Figure S6: a) Fluorescence spectra of dye (3.2 μM) titration with increasing concentrations of SC6 with inset showing the data fitting to an appropriate binding model; b) Spectral variation of dye (3.2 μM) and SC6 (0.70 mM) with increasing amounts of putrescine, at pH 7.2 (λ_{exc}=440 nm); c) Fluorescence spectra of dye (3.2 μM) titration with increasing concentrations of SC8 with inset showing the data fitting to an appropriate binding model; d) Spectral variation of dye (3.2 μM) and SC8 with increasing amounts of putrescine, at pH 7.6 (0.30 mM) (λ_{exc}=440 nm). The dashed line represents the free dye, the orange line represents the starting line of the changing trend, the gray lines the successive additions of SCn/putrescine, the light blue line the highest SCn:dye ratio (1:1), the dark blue line the highest SCn:dye ratio (1:2), the black line represents the dye:SCn, the green line the highest concentration of putrescine added, and the dashed line represents the free dye for comparison.

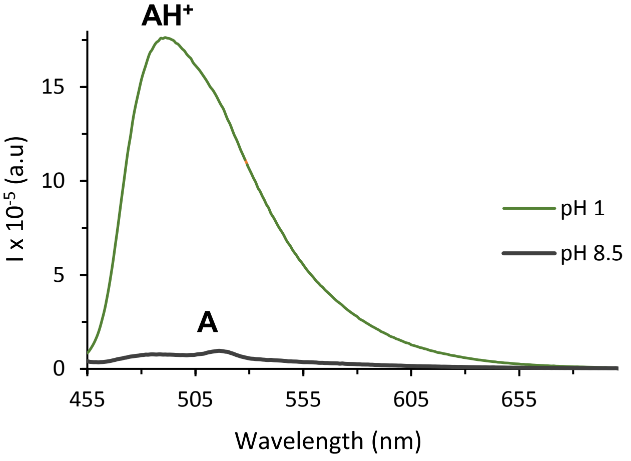

Figure S7: Fluorescence spectra of dye (3.2 μM) for the flavylium cation species (AH^+) recorded at pH 1 and the one for the neutral quinoidal base (A) recorded at pH 8.5 (λ_{exc}=440 nm).

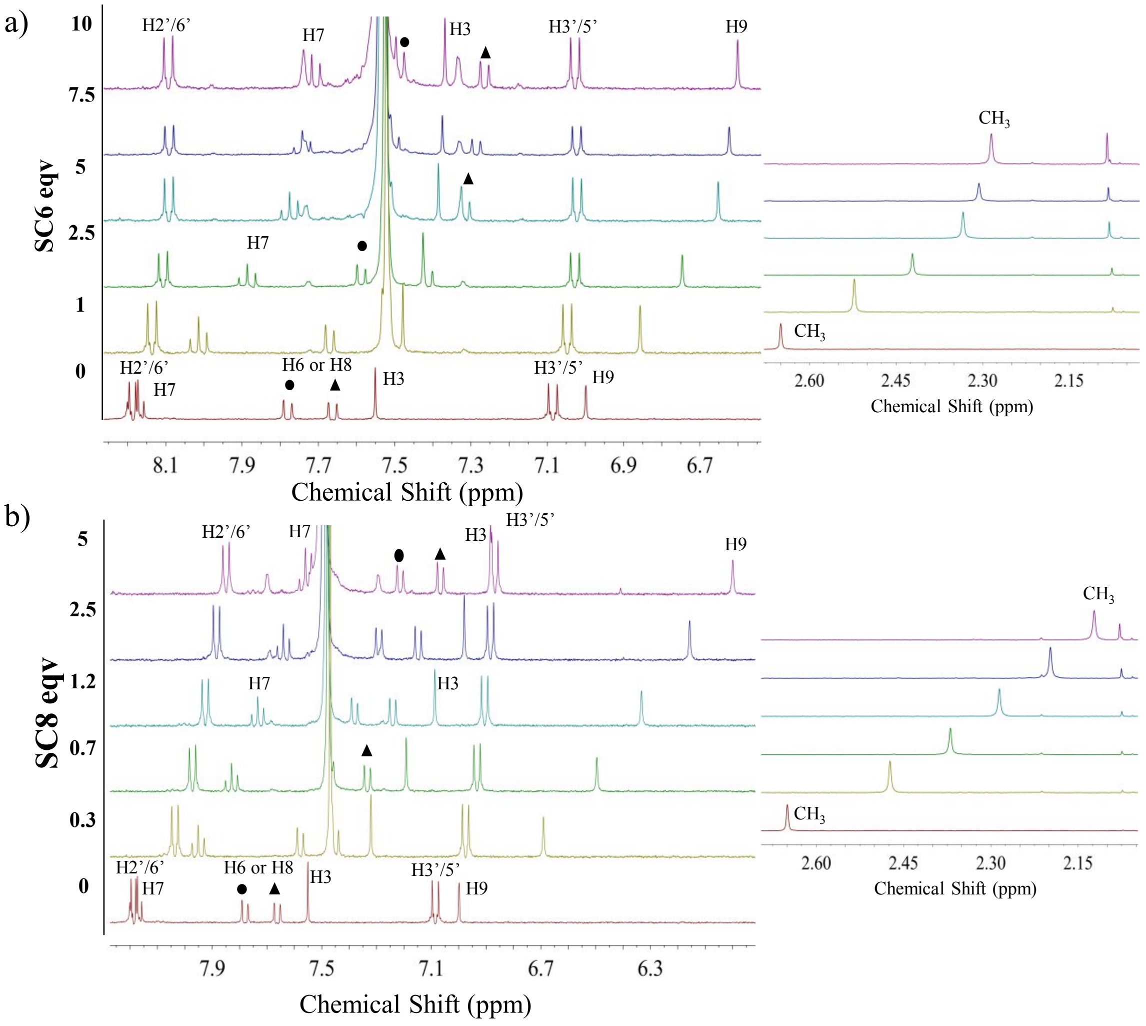

Figure S8: ^{1}H NMR spectra variations of dye (0.3 mM) solution upon titration with increasing concentrations of the host: a) SC6 and b) SC8. All spectra were acquired in D_2O/MeOD (80:20) at pH 1 with TSP as the internal standard. The protons were labelled according to Figure 3.
